# Furanodienone induces G0/G1 arrest and causes apoptosis via the ROS/MAPKs-mediated caspase-dependent pathway in human colorectal cancer cells: a study *in vitro* and *in vivo*

**DOI:** 10.1038/cddis.2017.220

**Published:** 2017-05-25

**Authors:** Ying Jiang, Xiaoqin Wang, Daode Hu

**Affiliations:** 1 Department of Clinical Pharmacology, Shanghai General Hospital, Shanghai Jiaotong University School of Medicine, Shanghai 200080, China

## Abstract

Furanodienone, a major bioactive constituents of sesquiterpene derived from *Rhizoma Curcumae*, has been proven to possess the potent anticancer efficacy on human breast cancer cells. Here, we investigated the cytotoxicity of furanodienone on human colorectal carcinoma cell lines *in vitro* and *in vivo*, as well as its underlying molecular mechanisms in the induction of apoptosis. In this study, we found that furanodienone significantly inhibited proliferation of RKO and HT-29 cells, induced mitochondrial dysfunction characterized by collapse of mitochondrial transmembrane potential and reduction of ATP level, and promoted the production of reactive oxygen species (ROS) that functions upstream of caspase-dependent apoptosis. The antioxidant *N*-acetyl cysteine, a ROS scavenger, abolished this apoptosis induced by furanodienone. In addition, furanodienone elevated the expression of p-p38, p-JNK, but decreased p-ERK, as a result of the produced ROS. The specific inhibitors U0126, SP600125 and SB202190 attenuated the expression of MAPKs, and regulated the expression of cleaved caspase-8, -9 and -3. Furthermore, the potential inhibitory effect of furanodienone on CRC cells was also corroborated in mouse xenograft model. In conclusion, the results demonstrated that furanodienone-triggered ROS plays a pivotal role in apoptosis as an upstream molecule-modulating activity of caspases in mitochondrial pathway via stimulating MAPKs signaling pathway. Our finding may provide a novel candidate for development of antitumor drugs targeting on colorectal cancer.

Colorectal cancer (CRC) is one of the commonest malignancy and the third leading cause of cancer-related deaths worldwide.^1^ In the United States alone, the incidence of CRC increases faster with an approximation of 134 490 new cases in 2016 in both sexes.^2^ At the same year in China, the new cases rose to 274 841, and the mortality was estimated 132 110 cases, which takes up 48% in colon-related death.^3, 4^ Although advancements in diagnosis and development of adjuvant therapy such as radiofrequency ablation, radiation therapy and chemotherapy for CRC have provided useful palliation,^5^ the 5-year relative survival rate from 2005 to 2011 was still 66% compared to that of 60% in the past decade.^2^ Furthermore, the survival rate tends to be 58.3% in the first 10 years after diagnosis.^6^ At present, the standard treatment modality for these patients is surgery in combination with chemotherapeutic regimens based on 5-Fluorouracil (5-FU) or leucovorin. However, the classic anticancer agents is frequently attenuated due to the development of resistance to chemotherapy.^7^ In addition, high-dose usage of these chemotherapeutic drugs did not improve the cure rate during the last decade and also brought considerable adverse side effects such as systemic toxicity. Therefore, there is an urgent need to screen novel drugs or metabolites that are capable of targeting on specific cancer.

Recently, there have been increasing attentions on exploration and application of natural plants owing to their merit of potent pharmacological activity.^8, 9, 10, 11, 12, 13^ Furanodienone is one of the major bioactive constituents derived from traditional Chinese medicine *Rhizoma Curcumae*.^14^ In our recent study, it was demonstrated that furanodienone possessed higher cytotoxic activity against CRC cells in comparison to other widely studied essential oils such as curdione, curcumol and germacrone from *R. Curcumae*.^15^ Although it was reported furanodienone inhibited MCF-7 cell proliferation by interfering with HER2 signaling pathway,^16, 17^ the possible molecular mechanisms of its inhibitory activity on human colon cancer cells have never been investigated.

It is well established that deregulation in the process of oxidative phosphorylation (OXPHOS) has a key role in cells fate. Reactive oxygen species (ROS) majorly originated from OXPHOS regulate cells growth and the redox status, affecting biochemical processes. Actually, ROS have a dual effect on maintaining the normal physiological activities within cells. Under normal conditions, a moderate level of ROS is well-controlled to modulate apoptosis, proliferation and inflammatory responses, contributing to a beneficial environment for cells survival.^18, 19^ However, ROS in a specific condition, especially when the level of free radicals far exceeds its scavenging ability in cells, can damage cellular components such as DNA, protein and lipids, thus interfering with cellular signaling pathways. Excessive accumulation of ROS has been reported to amplify death execution through redox-sensitive signaling by disrupting the expression of several apoptotic effectors such as Bcl-2, caspases and cytochrome *c* in the mitochondrial-mediated apoptotic pathway,^20^ which provides a train of thoughts to develop redox-modifying drugs.

ROS generation involved increasingly in cell proliferation and apoptosis via regulation of MAPK family members comprising of ERK, JNK and p38 MAPK in the action of some chemotherapeutic drugs.^21, 22^ MAPKs play an essential role in diverse cellular programs including modulation the pro and anti-apoptotic proteins located in mitochondria.^23^ For example, in response to oxidative stress, the activation of JNK promotes the apoptotic processes, in a manner of inhibition of Bcl-2 function.^24^ Furthermore, degradation of Bcl-2 was found to associate with downregulation of ERK when exposed to oxidative stress.^25^ Accordingly, targeting activation of the ROS/MAPK signaling pathway may be a promising strategy for enhancement of antitumor efficacy in the treatment of human cancers.

The aim of the present study was to characterize the cytotoxic effects and molecular mechanisms of furanodienone on RKO or HT-29 colon cancer cells *in vivo* and *in vitro*. Collectively, it was showed for the first time the evidence that furanodienone induces G0/G1 phase arrest and causes apoptosis depending on the production of ROS that activates MAPKs signaling pathway in a caspase-dependent manner in RKO and HT-29 cells.

## Results

### Furanodienone inhibits colony formation and induces cell cycle G0/G1 arrest

**Figure 1:**
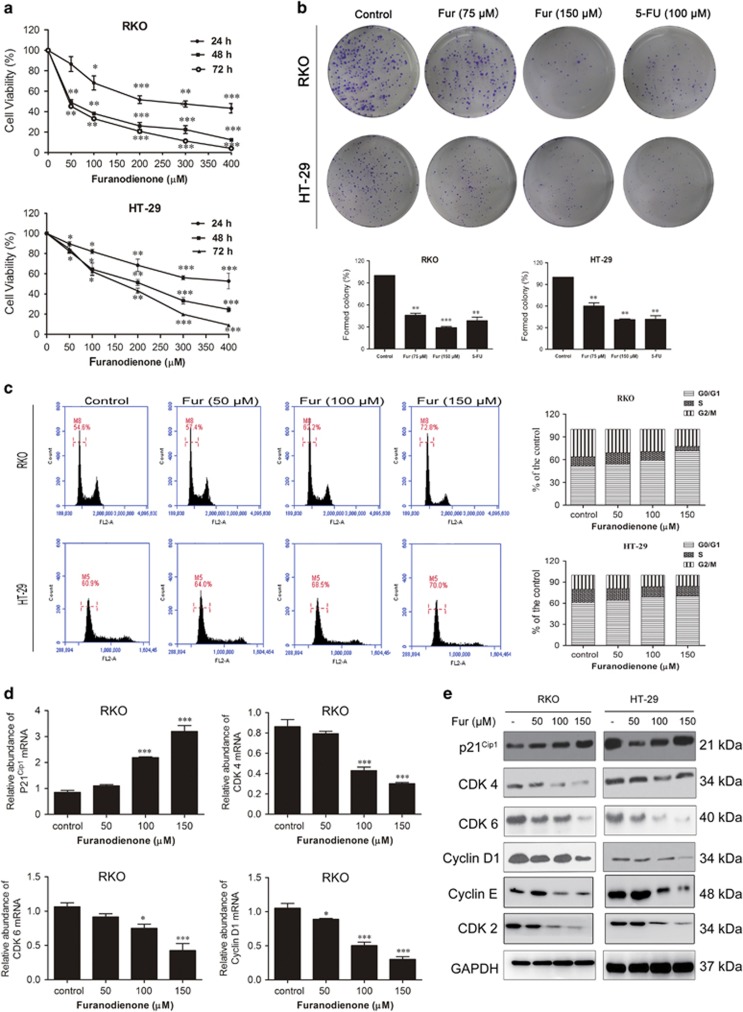
Furanodienone inhibits cells proliferation and induces G0/G1 arrest in human colorectal cancer cells. (**a**) The antitumor effect of furanodienone on RKO and HT-29 cells was measured by CCK-8 assay. Cells were treated with raising doses of furanodienone for 24, 48 and 72 h. Each experiment performed in triplicate. (**b**) Colony formation assay of RKO and HT-29 cells with control, furanodienone or 5-FU. (**c**) Furanodienone induced G0/G1 phase arrest. RKO and HT-29 cells were exposed to furanodienone (0, 50, 100 and 150 *μ*M) for 24 h followed by flow cytometric assay. The percentage of cell cycle distribution was showed as mean±S.D. from three independent experiments. The mRNA expression of p21^Cip1^, CDK 4, CDK 6 and cyclin D1 in RKO cells treated with furanodienone for 24 h were detected using RT-qPCR (**d**). The expression of G0/G1 phase-related proteins were analyzed by western blotting (**e**). *P<0.05, **P<0.01, ***P<0.0001, significantly different compared with control

Five types of CRC cells were used to evaluate the death-inducing activity of furanodienone, including RKO (poorly differentiated cell), sw480 (poorly differentiated cell), HT-29 (intermediate-differentiated cell), sw620 (intermediate-differentiated cell) and LoVo (well-differentiated cell). As shown in Supplementary Figure S1a, the IC_50_ in 24 h were, respectively, 156.4 *μ*M (RKO), 73.7 *μ*M (sw480), 251.1 *μ*M (HT-29), 412.5 *μ*M (sw620) and 573.8 *μ*M (LoVo), while that in 48 h were 51.8 *μ*M (RKO), 44.18 *μ*M (sw480), 168.9 *μ*M (HT-29), 314.2 *μ*M (sw620) and 502.1 *μ*M (LoVo), respectively. Furanodienone inhibits proliferation on all of these CRC cells, indicating the inhibitory effects of furanodienone are cell line-independent. RKO and HT-29 as respective representative of poorly differentiated and intermediate-differentiated CRC cells were taken as our studied objects. As shown in Figure 1a, RKO and HT-29 cells viability were both inhibited in a time- and dose-dependent manner. The IC_50_ for 5-FU (Supplementary Figure S1b) in 24 h was calculated as 67.4 *μ*M (RKO) or 85.2 *μ*M (HT-29), showing a bit stronger in cytotoxicity compared to furanodienone. Additional colony formation assay was conducted to confirm the antiproliferative effect of furanodienone on these two colon cancer cells (Figure 1b). The formed colonies in both cells were significantly reduced with the raising concentrations of furanodienone. While compared with 5-FU, furanodienone at 150 *μ*M showed similar cytotoxic effects on RKO and HT-29 cells, indicating that furanodienone can be regarded as a potential drug targeting CRC.

To dissect whether the cells inhibition induced by furanodienone involves in cell cycle arrest, cell cycle distribution of both cells was evaluated using flow cytometry, and the expression of cell cycle regulatory proteins was analyzed by RT-qPCR and western blotting subsequently. The results in Figure 1c showed that furanodienone significantly increased cell number at G0/G1 phase and led to a corresponding decrease in G2/M phase compared to the control, however, there was no obvious effect on S phase in both cells. RT-qPCR results showed that mRNA expression of the key related checkpoint factors in G0/G1 phase including p21^Cip1^ was significantly upregulated in RKO cells in a dose-dependent manner, whereas mRNA expression of cyclin D1, CDK 4 and CDK 6 was decreased (Figure 1d), in accordance with the results from western blotting (Figure 1e). Besides, CDK 2 and cyclin E protein levels in both cells were downregulated (Figure 1e). All these data manifested that G0/G1 phase arrest may be accounted to the furanodienone-induced antiproliferation against RKO and HT-29 cells.

### Furanodienone induces apoptosis of colorectal cells

**Figure 2:**
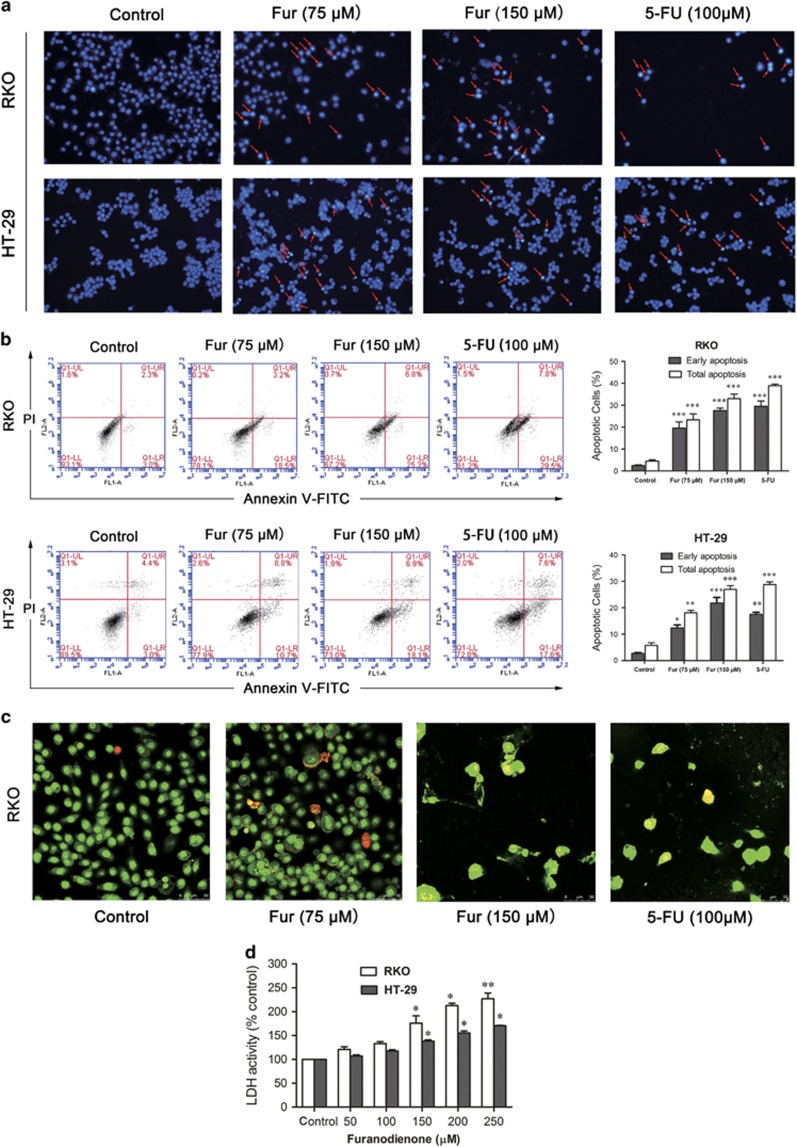
Furanodienone induces apoptosis in CRC cells. (**a**) Apoptotic nuclear morphology changes induced by furanodienone or 5-FU were assessed by DAPI staining and visualized by fluorescence microscopy at a magnification of × 200. Red arrows indicate chromatin and nuclear fragmentation. (**b**) Cells were treated with raising concentrations of furanodienone or 100 *μ*M 5-FU for 24 h. The apoptotic rates were measured and analyzed by flow cytometry using Annexin V-FITC/PI staining. Each experiment performed in triplicate. (**c**) Apoptotic cells treatment with furanodienone (75 and 150 *μ*M) or 5-FU (100 *μ*M) for 24 h were imaged using AO/EB staining at a magnification of × 400. Cells exhibiting yellow or red fluorescence indicated apoptosis or necrosis. (**d**) Dose-dependent induction of LDH leakage to cells after furanodienone exposure for 24 h. *P<0.05, **P<0.01, ***P<0.0001, significantly different compared with control. Each experiment performed in triplicate

Apoptosis can be regarded as one of the critical molecular mechanisms in drug-induced cell death. To figure out if apoptosis is responsible for the cells inhibition triggered by furanodienone, we performed DAPI staining. Cells stained with DAPI presented brighter blue fluorescence in high-dose group compared with the control group in both cells (Figure 2a). As in the flow cytometric analysis, there were significant increases with an elevation of furanodienone in a dose-dependent manner in both early and late apoptosis, while the addition of 75 and 150 *μ*M furanodienone increased the apoptotic rates to 19.45±2.37% and 27.34±0.79%, respectively, compared with 2.34±0.45% in control group in RKO cells, whereas 12.4±1.08 and 20.64±3.02% apoptosis at the concentration of 75 and 150 *μ*M furanodienone were observed in HT-29 cells compared with 2.89±0.26%. 5-FU in 100 *μ*M showed a similar proapoptotic effect on both cells compared with that in 150 *μ*M furanodienone. AO/EB staining assay confirmed the apoptotic characterizations in furanodienone-treated RKO cells (Figure 2c), consistent with a raising level in LDH release with an increase in furanodienone concentration for 24 h (Figure 2d). Thus, the overall study above suggested that furanodienone induced apoptosis in RKO and HT-29 cells.

### Furanodienone induces cells apoptosis through caspase-dependent extrinsic and intrinsic pathways

**Figure 3:**
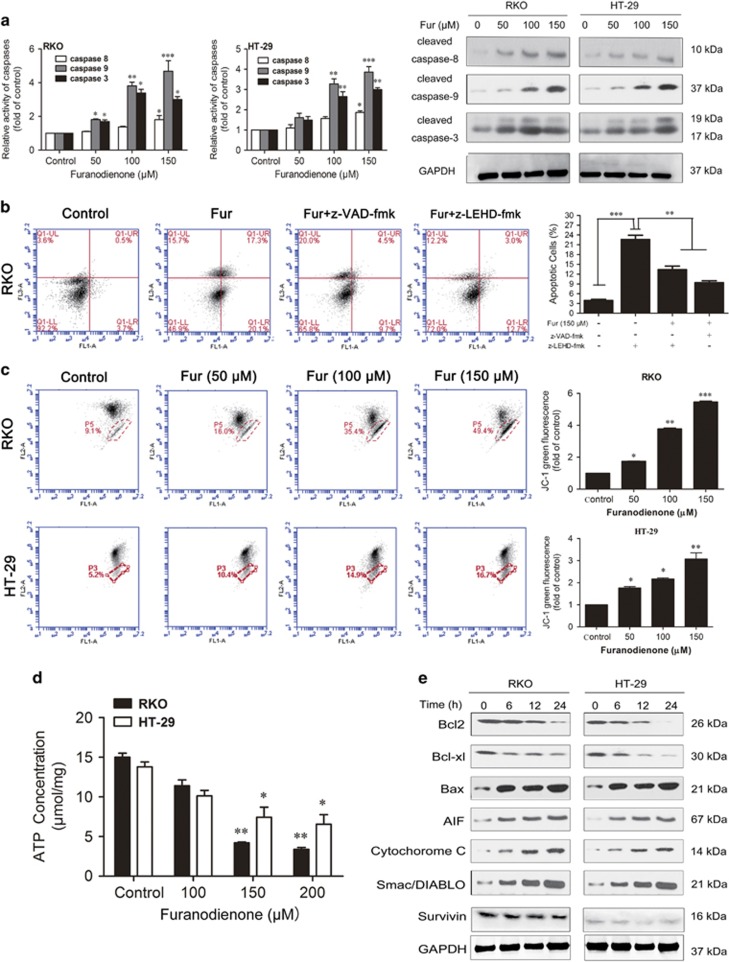
Furanodienone-induced apoptosis involves mitochondrial pathway in CRC cells. (**a**) Activity of caspase-8, -3 and -9 in both cells after exposure to furanodienone were detected using activity assay, and the expression of cleaved caspase-8, -3 and -9 proteins were measured using western blot. (**b**) Pretreatment with the general caspases inhibitor z-VAD-fmk or the specific caspase-9 inhibitor z-LEHD-fmk reserved apoptosis in RKO cells after exposure of 150 *μ*M furanodienone. Cells were pretreated with 100 *μ*M z-VAD-fmk or 100 *μ*M z-LEHD-fmk for 2 h before exposed to furanodienone. The apoptotic rates were detected using flow cytometry. (**c**) The mitochondrial membrane potential were investigated using JC-1 staining. Results indicating intensity of green fluorescence showed as folds of control from three independent experiments. (**d**) Twenty-four hours after furanodienone treatment, ATP concentration was measured. The results were presented as the mean±S.D. of triplicate experiments. (**e**) Proteins in mitochondrial pathway were analyzed using western blotting. *P<0.05, **P<0.01, ***P<0.0001, significantly different compared with control

Apoptosis can be induced either by extrinsic pathway through the activation of cell surface death receptors or by intrinsic pathway through the release of mitochondria–related signal factors,^26, 27^ while activation of caspase-8 and caspase-9 is required, respectively. To determine which pathway associated with the furanodienone-induced apoptosis, we detected the activity of caspase-8, -3 and -9. As shown in Figure 3a, an obvious increase in caspase-9 and -3 activity has been observed in both cells, whereas a relative minor effect on that of caspase-8. Pretreatment with a specific caspase-9 inhibitor (z-LEHD-fmk) partially reversed the apoptosis in RKO cells (Figure 3b), while the general caspases inhibitor (z-VAD-fmk) exhibited a similar effect with that of z-LEHD-fmk, thus we suspected furanodienone-induced apoptosis involves mainly in the intrinsic pathway. To confirm the involvement of mitochondrial pathway in induction of apoptosis, changes of the mitochondrial membrane potential (*Δ*ψ_*m*_) were tested with fluorescent mitochondrial probe JC-1. Flow cytometric analysis in Figure 3c showed there was a significant loss in *Δ*ψ_*m*_, which was evidenced by accumulations of cells stained with green fluorescence after exposure to furanodienone. The relative ATP level investigation showed a statistically significant drop in intracellular ATP levels compared to the control (Figure 3d). Moreover, following treatment with furanodienone, the expression of Bax, cytochrome *c*, Smac/DIABLO and AIF proteins were obviously increased and, in contrast, Bcl-2, Bcl-xl and survivin were decreased in a time-dependent manner (Figure 3e), implying activation of the intrinsic apoptosis pathway in furanodienone-induced cell death. Taken together, the apoptosis induced by furanodienone was dependent on caspase-dependent intrinsic and extrinsic signaling pathways, mainly by the intrinsic apoptotic pathway.

### Intracellular ROS generation involves in mitochondria-mediated cell death in furanodienone-treated cells

**Figure 4:**
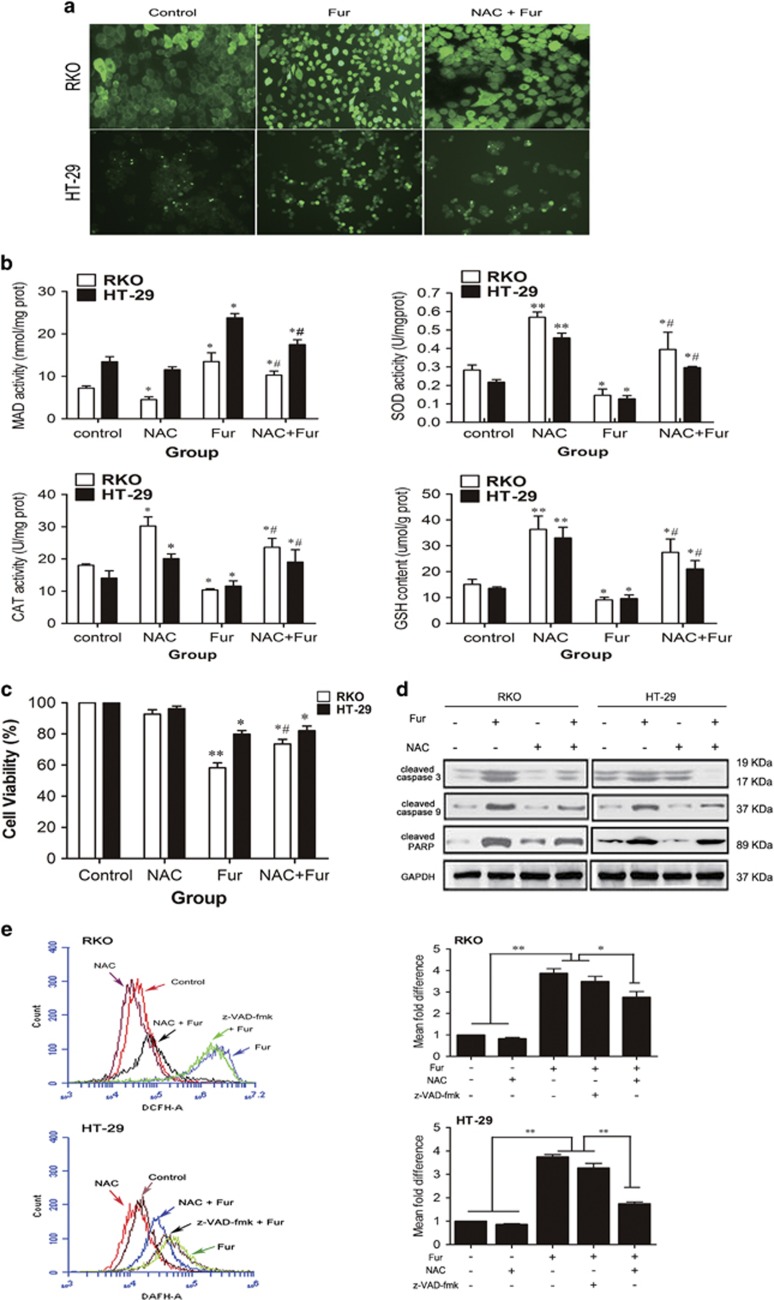
Furanodienone increases ROS production that actions upstream of the mitochondrial pathway. (**a**) Fluorescence images of cells were observed using DCFH-DA probe at a magnification of × 100. Cells were pretreated with or without 10 mM NAC for 2 h, followed by 150 *μ*M furanodienone exposure for 24 h. (**b**) Changes of MDA level, SOD and CAT activity, and GSH content at 24 h in furanodienone-treated cells. After exposure to 150 *μ*M furanodienone plus pretreatment with or without 10 mM NAC for 2 h, the production of MDA, activity of SOD and CAT, and GSH content were measured. Date are expressed as mean±S.D. from three independent experiments. (**c**) Cells were preincubated with 10 mM of NAC for 2 h before treatment with 150 *μ*M of furanodienone for 24 h, and the cell viability was measured by CCK-8 assay. (**d**) In the absence or presence of NAC, the cleaved caspase-3, -9 and PARP cleavage proteins exposure of 150 *μ*M furanodienone were quantified by western blotting. (**e**) Total ROS was determined using DCFH-DA probe (10 *μ*M). Cells pretreatment with 10 mM NAC or 100 *μ*M z-VAD-fmk for 2 h were then treated using 150 *μ*M furanodienone for 24 h, and ROS level was measured by flow cytometry. *P<0.05, **P<0.01 *versus* control, ^#^*P*<0.05 for Fur *versus* NAC+Fur

ROS is verified a pivotal role in the activation of cell death pathways. We next explored if the apoptotic effect of furanodienone was a function of ROS modulation within cells. The change of ROS levels in both cells after exposure to furanodienone was measured by DAFH-DA probe. As illustrated in Figure 4a, furanodienone markedly increased ROS production, while *N*-acetyl cysteine (NAC) attenuated it. MDA as one of the symbolic markers of oxidative stress was observed to be enhanced by twofold in furanodienone group, whereas the activity of SOD and CAT was downregulated, together with the content of GSH (Figure 4b). However, treatment with NAC remarkably reversed the elevated MDA level, while the activity of SOD and CAT, and GSH level were upregulated. CCK-8 analysis in Figure 4c further suggested that NAC abolished furanodienone-induced apoptosis, and the expression of cleaved caspase-3, -9 and cleaved PARP in mitochondrial pathway was reduced in the presence of NAC (Figure 4d). Thus, there is a reason to believe that ROS involves in the furanodienone-induced apoptosis via activating the mitochondrial apoptotic pathway. To elucidate how ROS functions in mitochondrial pathway, cells preincubated with the general caspases inhibitor z-VAD-fmk were analyzed by flow cytometry. ROS production was explosively increased at 150 *μ*M furanodienone within 24 h, but a significant reduction was observed on a pretreatment of NAC, whereas it remained unchanged in inhibitor group compared with that in the furanodienone group (Figure 4e), which indicated that ROS is a upstream molecule-modulating caspase-dependent reaction. The results above suggested that the antitumor activity of furanodienone was a function of mitochondria-apoptotic execution secondary to the modulation of ROS.

### Furanodienone induces apoptosis via activating MAPKs-mediated mitochondrial pathway dependent of ROS production

**Figure 5:**
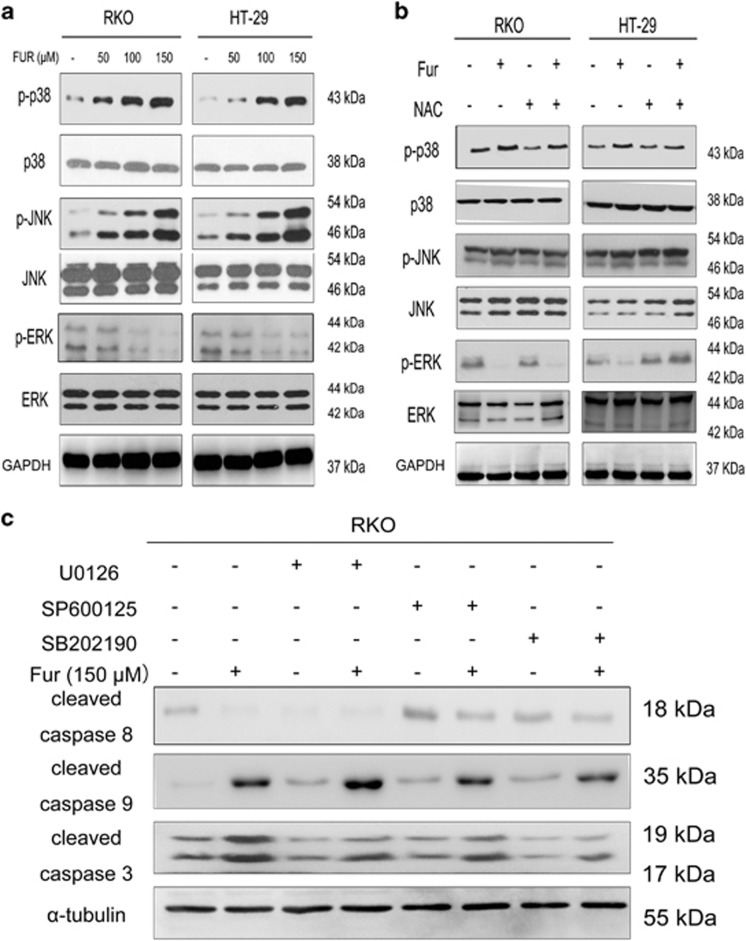
The produced ROS contributes to the MAPKs-mediated mitochondrial pathway in apoptosis induced by furanodienone. (**a**) The protein expressions of p-p38, p38, p-JNK, p-JNK, p-ERK and ERK were measured by western blotting. Cells exposed to varying concentrations of furanodienone (50, 100 and 150 *μ*M) were detected for the expression of MAPKs proteins. (**b**) Regulation of ROS/MAPKs-dependent mitochondrial pathway. The expressions of p-P38, P38, P-JNK, JNK, P-ERK and ERK were measured by western blot assay after 24 h of exposure to furanodienone (150 *μ*M) plus NAC (10 mM) in both cells. (**c**) RKO cells were pretreated with or without 20 *μ*M U0126, SP600125 or SB202190 for 1 h followed by furanodienone (150 *μ*M) treatment for an additional 24 h. Expressions of cleaved caspase-8, -9 and -3 were determined by western blotting

The possible interlink between oxidative stress and MAPKs pathway in RKO and HT-29 cells were examined by western blotting. Furanodienone significantly induced the phosphorylations of p38 and JNK in a dose-dependent manner, and unexpectedly, the expression of p-ERK was reduced (Figure 5a). The antioxidant NAC reduced p-p38, p-JNK and increased p-ERK levels in Figure 5b. However, expression of p38, JNK and ERK remained unchanged. We further illuminated the relationship between MAPKs and furanodienone-induced caspase-dependent apoptosis. RKO cells were pretreated with three specific inhibitors, respectively, U0126 (an ERK inhibitor), SP600125 (a JNK inhibitor) and SB202190 (a p38 inhibitor) for 2 h, and then analyzed by western blotting. As shown in Figure 5c, SP600125 and SB202190 significantly inhibited the expression of cleaved caspase-8, -9 and -3, while U0126 exhibited an opposite trend. These results suggested that furanodienone-induced ROS activated MAPKs signaling pathway, which further elaborated the mitochondria-mediated apoptosis via modulating the caspase-dependent pathway.

### Furanodienone inhibits tumor growth in a xenograft murine model

**Figure 6:**
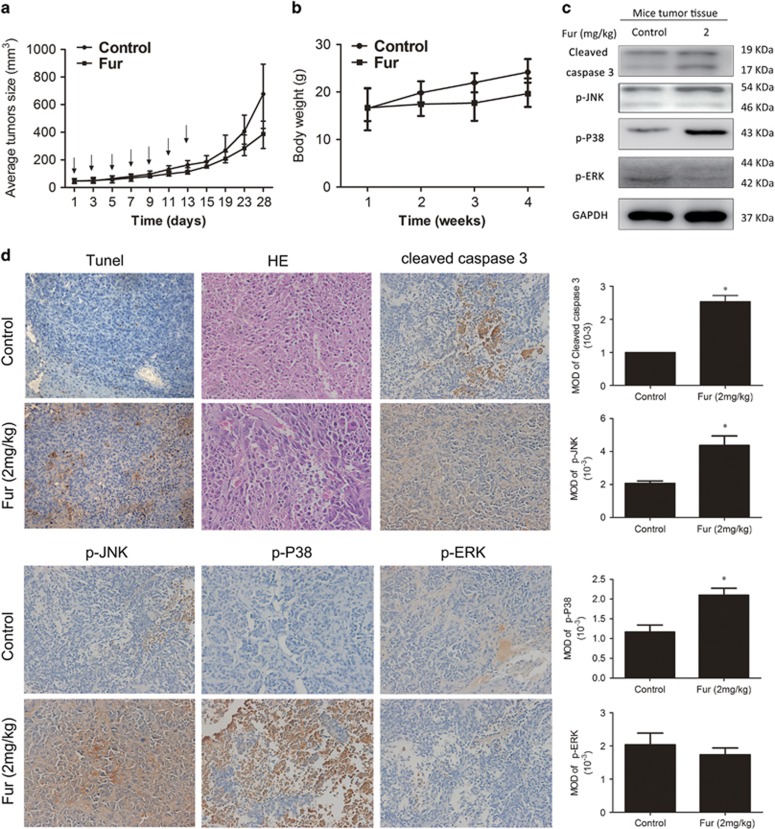
Furanodienone inhibits tumorigenesis of human colorectal xenograft *in vivo*. RKO cells were inoculated subcutaneously into the right flank of BALB/c-nu. When the average size of tumors reached 50 mm^3^, mice were randomly divided into two groups receiving intraperitoneal injections with 10% DMSO or furanodienone (2 mg/kg) every other day during 2 weeks. (**a** and **b**) Tumor sizes and body weights were measured on a weekly basis. (**c**) The level of cleaved caspase-3, p-JNK, p-P38 and p-ERK in tumor xenograft tissues was detected by western blot. (**d**) The apoptotic status in tumor tissues was assessed by TUNEL assay. HE staining was used to evaluated the histology. The expression of cleaved caspase-3, p-JNK, p-P38 and p-ERK was examined by immunohistochemistry, and mean optical density of which were quantified by Image Pro-Plus (IPP, Media Cybernetics, BD Biosciences, Mountain View, CA, USA). *P<0.05, significantly different compared with control

We established the BALB/c-nu models by transplanting RKO cells subcutaneously into the right flank of mice to confirm the potential inhibitory effect of furanodienone on CRC cells *in vivo*. When the tumor size reached 50 mm^3^, the mice were divided into two groups: control (10% DMSO) and furanodienone (2 mg/kg), followed by intraperitoneally injection every other day for seven times. The average size of the tumors in 2 mg/kg furanodienone-treated group was reduced to 374.4 mm^3^ compared to 648.2 mm^3^ in 10% DMSO-treated group. Furanodienone significantly inhibited the growth of tumor size, but exerted a minor effect on its body weight (Figures 6a and b). As results presented in Figure 6c, furanodienone increased level of cleaved caspase-3, p-P38 and p-JNK, but reduced that of the p-ERK. TUNEL-positive cells were significantly increased in experiment group, and HE staining assay suggested that furanodienone has no overall toxicity on tissues (Figure 6d). Immunohistochemistry showed positive stains in cleaved caspase-3, p-P38 and p-JNK, while a negative in p-ERK, which was quantified by IPP software in terms of mean optical density (Figure 6d). All these results revealed that furanodienone inhibits growth of CRC cells *in vivo* with low toxicity.

## Discussion

*R. Curcumae*, a member of the genus Curcuma, is cultivated in tropical and subtropical countries, especially in China, and the extracted sesquiterpene volatile oils possess powerful anticancer properties.^28, 29^ Furanodienone, a main bioactive constituent in sespuiterpenes from *R. Curcumae*, has been reported the potential antitumor potent against human breast cancer cells.^17^ Our previous study found that furanodienone exerts the best inhibitory effect on the proliferation of CRC cells among other three essential oils, implying that furanodienone may have a positive value in treatment of CRC.^30^ However, the cytotoxic effects and its underlying molecular mechanisms have not been elaborated. In this study, the potential cytotoxic effects of furanodienone on CRC were comprehensively investigated using both *in vitro* and *in vivo* models. Our results for the first time presented that furanodienone induced G0/G1 cell cycle arrest and caused apoptosis.

Anticancer effect is usually mediated by the inhibition of proliferation and cell cycle arrest. Cell cycle deregulation is one of the hallmarks in tumor cells and mutations in key checkpoint genes, especially the family of cyclin-dependent kinase (CDK), contributing to tumor-associated cell cycle defects.^31^ The progression of cell cycle is driven by different cyclin-CDK complexes via phosphorylating the target proteins. CDK 4 and CDK 6 are essential in the progression of G1 phase by forming the CDK 4/6-cyclin D1 complexes, while cyclin E and CDK 2 were necessary in the late of G0/G1 cell phase.^32, 33^ CDK inhibitor, p21^Cip1^, has been reported to be related with the G0/G1 phase arrest by inactivation of CDK-cyclin complex (CDK 4/cyclin D and CDK 2/cyclin E).^32^ Consistent with results from the previous study,^16^ our study reflected that furanodienone increased the proportion of G0/G1 phase, and reduced the cell population in G2/M phase in RKO and HT-29 cells, according to the flow cytometric analysis. Further RT-qPCR revealed that cyclin D1, CDK 4 and CDK 6 mRNA expressions were reduced, whereas p21^Cip1^ mRNA was increased in RKO cells. In addition, furanodienone led to a decrease in accumulation and activation of G0/G1 phase-related cycle regulator. Thus, the reduction in level of CDK 4, CDK 6, cyclin D1, CDK 2 and cyclin E proteins and upregulation in p21^Cip1^ may be explained for G0/G1 phase arrest induced by furanodienone.

Apoptosis (or type-I programmed cell death), firstly put forward by Keer in 1972,^34^ was recognized as a physiological process that is characterized by a wide range of pathological conditions or morphological changes such as cell shrinkage, chromatin condensation, cellular fragmentation and plasma membrane blebbing.^35, 36^ It was widely accepted that apoptosis can be stimulated through two major apoptotic pathways: the extrinsic cell surface death receptor-directed apoptotic pathway and the intracellular sensor-mediated apoptotic pathway, and both of which involve in the activation of caspases that are usually expressed in an inactive proenzyme form before being stimulated. Once activated, the caspases initiate the downstream pro-caspases followed by the activation of protease cascade.^37^ Caspase-8 as an apical caspase and caspase-9 as an important intracellular amplifier of caspase signaling downstream of mitochondria are, respectively, indispensable in the extrinsic and intrinsic apoptosis pathway. Present study found that furanodienone-treated cells activated caspase-3, -8 and -9. Besides, the cleaved caspase-3 level in tumor tissues treated with furanodienone was confirmed by immunohistochemical analysis (Figure 6d). The proapoptotic members (Bax, Bad and Bak) of Bcl-2 family that regulates the mitochondrial outer membrane permeabilization initiate the release of cytochrome *c*, Smac/DIABLO and AIF into the cytosol, however, Bcl-2 and Bcl-xl as anti-apoptotic members antagonize them by heterodimerising with proapoptotic proteins.^38^ In addition, ATP depletion and MMP collapse as signs of mitochondrial dysfunction, all account for the ultimately mitochondrial damages.^39^ In the present study, decrease in the Bcl-2/Bax ratio has been detected by western blotting, companying with the increasing accumulations of cytochrome *c*, Smac/DIABLO and AIF in the cytosol. Meanwhile, declines of MMP and ATP induced by furanodienone were recorded as well. Thus, we can draw the conclusion that mitochondria-mediated apoptotic pathway was involved in the furanodienone-induced cell apoptosis.

ROS as an important molecule takes part in regulations of cell death and survival in cancer cells. We confirmed furanodienone triggered excessive ROS production. As a result, the produced ROS elevated the level of MDA, which is one of the final products of lipid oxidation by oxidative stress. Meanwhile, activity of antioxidant enzymes (SOD and CAT) was inhibited by ROS, and the GSH content were reduced, which is consistent with some of the previous reports indicating an imbalance in oxidant/antioxidant system induced by oxidative stress.^40, 41^ This may be explained by the inhibition of nuclear factor-erythroid-2-related factor 2 (Nrf2) signaling. Nrf2, a redox-sensitive basic-leucine zipper transcription factor, is essential for the cellular defense responses via transactivating genes of a large battery of antioxidant enzymes and phase II detoxifying enzymes to alleviate the burden of oxidative stress.^42, 43^ The SOD and CAT are downstream genes induced by Nrf2 translocation and their activities were enhanced as adaptive intracellular responses to protect against oxidative damages.^44, 45^ However, when the produced ROS cannot be eliminated in time, Nrf2 expression is counteracted by the activation of p53 in response to substantial generation of oxidative stress, which might account for the attenuated SOD and CAT activity.^46^ NAC as a source of GSH has a potent ability to promote the antioxidative system and the activation of the Nrf2 signaling. Pretreatment with NAC, the changes in MDA level, SOD or CAT activity, and GSH content were reversed. Furthermore, the activated Nrf2 facilitates the production of GSH. In the present study, the furanodienone-induced apoptosis potential was clearly a result of oxidative imbalance as indicated by enhanced level of ROS since NAC pretreatment abolished apoptosis induced by furanodienone (Figure 4c). Moreover, pretreatment with NAC significantly decreased caspase-3 and -9 expressions; in contrast, the level of cleaved PARP was upregulated. Furthermore, in accordance with a study conducted by Maillet *et al.*,^47^ ROS as a upstream molecule regulates caspases’ activation, indicating ROS involved in the mitochondrial pathway.

**Figure 7:**
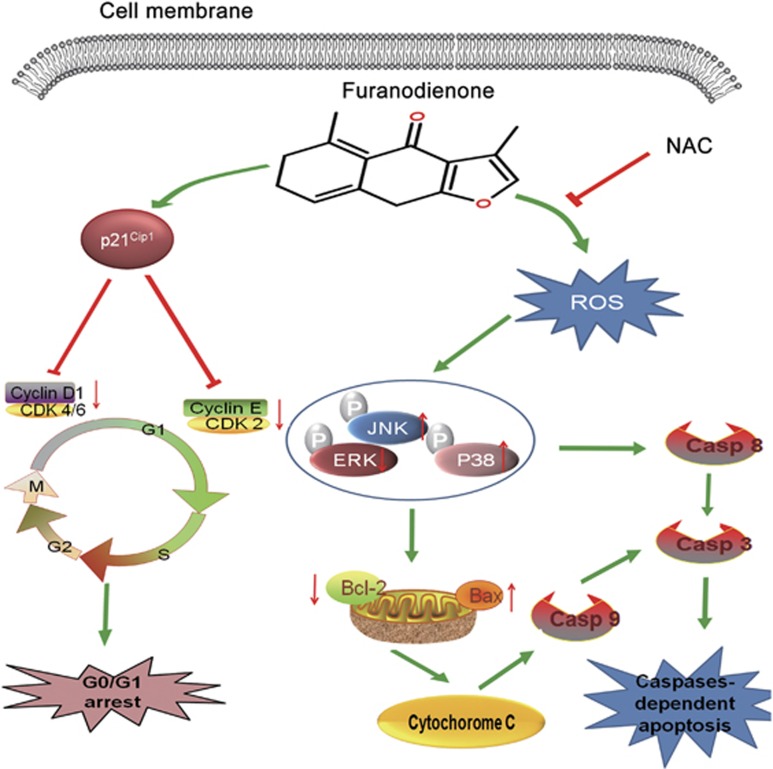
A schematic diagram of G0/G1 phase arrest and ROS-dependent MAPKs pathway in which furanodienone induces cell growth inhibition and cell death in CRC cells

Growing evidence in recent years demonstrates the possible interdependent relationship between the MAPKs pathway and oxidative stress in apoptosis. MAPKs are important mediators playing a pivotal role in modulating the actions of related mitochondrial proteins, including ERK1/2, JNK and p38. Generally speaking, the activation of ERK1/2 promotes cell proliferation, opposing the proapoptotic functions exerted by the stress-activated JNK and p38 MAPK pathways.^48, 49^ In the present study, we found there was a significant increase in JNK and p38 phosphorylation, while reduced in ERK1/2 phosphorylation when treated with furanodienone. Pretreatment with NAC reserved the furanodienone-induced JNK and p38 MAPK phosphorylation (Figure 6b). Previous reports have shown that activation of MAPKs signaling pathway modulates the expression of pro or anti-apoptotic proteins (Bax and Bcl-2) translocated onto mitochondria dependent of the production of ROS, which initiates the mitochondrial-derived apoptosis.^23, 50^ Our study showed that JNK and p38 inhibitors reversed the furanodienone-induced increasing expressions of cleaved caspase-8, -9 and -3, while ERK1/2 inhibitor showed an opposite trend, indicating MAPKs participated in the mitochondrial apoptotic pathway, but whether the apoptosis regulates the activity of pro or anti-apoptotic proteins should be further studied. Taken together, our results illustrated that furanodienone triggered apoptosis through activation of ROS-dependent MAPKs-mediated caspase-dependent pathway (Figure 7).

In conclusion, our study for the first time identifies furanodienone-induced antitumor effects on CRC cells *in vitro* and *in vivo*. We found that furanodienone significantly induced G0/G1 phase arrest and caused cell apoptosis via ROS-dependent MAPKs-mediated mitochondrial pathway. We also demonstrated that furanodienone significantly decreased tumor growth in BALB/c-nu mice bearing xenografts. Overall, furanodienone can be regarded as a leading compound to explore a potential safe drug with low toxicity targeting on CRC. Moreover, this study provided insight into molecular mechanisms on furanodienone-induced cell death, which may aid the development of clinical usage of a novel drug against CRC.

## Materials and methods

### Reagents, chemicals and antibodies

Furanodienone (⩽98%) and 5-FU were purchased from Shanghai Yuanye Bio-Technology Co., Ltd, (Shanghai, China). A stock solution of furanodienone at 4 mM was prepared in dimethyl sulfoxide (DMSO; Sigma, St. Louis, MO, USA) and stored at −20 °C, while that of 5-FU was prepared in phosphate-buffered saline (PBS) and stored at 4 °C. Dulbecco’s modified Eagle’s medium (DMEM) and 1 × (PBS, pH 7.4) were obtained from Jinuo Biotechnology (Hangzhou, China). Caspase-3, -8 and -9 Activity Assay Kits, Reactive oxygen species Assay Kit, BCA protein assay Kit and Cell Cycle and Apoptosis Analysis Kits were purchased from Beyotime Biotechnology (Suzhou, China). The NAC was purchased from Sigma Chemical Co. (St. Louis, MO, USA), and inhibitors z-VAD-fmk, z-LEHD-fmk, U0126, SP600125 and SB202190 were afforded by Shanghai Qiangzhi Bio-Technology Co., Ltd, (Shanghai, China). The primary antibodies: p21^Cip1^, CDK 4, cyclin D1, CDK 6, CDK 2, cyclin E, caspase-8, -3 and -9, cleaved caspase-8, -3 and -9, cleaved PARP, Bax, Bcl-2, Bcl-xl, cytochrome *c*, AIF, Smac/DIABLO, survivin, p-p38, p38, p-JNK, JNK, p-ERK, ERK, *α*-tubulin and GAPDH were afforded by Cell Signaling Technology (Beverly, MA, USA).

### Cell culture

Human colon adenocarcinoma cells were gifted from the Institute of Clinical Translational Research, Shanghai General Hospital (Shanghai, China). All of these cells were incubated in high-glucose DMEM supplemented with 10% fetal bovine serum (Gibco Laboratories, Shanghai, China) and 1% penicillin/streptomycin (100 *μ*g/ml for streptomycin, 100 U/ml for penicillin; Gibco Laboratories) at 37 °C in a 5% CO_2_ humidified atmosphere.

### Cell proliferation assessment by CCK-8 assay

Cell proliferation was determined with the CCK-8 assay (Dojindo, Tokyo, Japan). To evaluate the antiproliferative effect of furanodienone on human CRC cells, cell suspensions (8 × 10^4^/ml) were seeded in 96-well plates in growth medium, overnight. Cells were treated with various concentrations of furandienone (50, 100, 150, 200, 250, 300, 350 and 400 *μ*M) and the group treated with 0.1% DMSO was taken as control. At appropriate time points (i.e., 24, 48 or 72 h), 90 *μ*l fresh medium was incubated with 10 *μ*l CCK-8 solution in each well for 2 h at 37 °C before absorbance was read at 450 nm wavelength using a microplate reader (Bio-Tek, San Jose, CA, USA).

### Clone formation assay

Cells at a density of 500 cells per well were evenly dispersed on six-well plates followed by furanodienone (75 and 150 *μ*M) or 5-FU (100 *μ*M) treatment for 10 days. After washing with PBS, the visible colonies were fixed for 30 min in 4% paraformaldehyde and stained with crystal violet for 20 min at room temperature. Then the counts of established cell colonies >50 were manually scored and the images were recorded by a digital camera.^12^

### Cell cycle analysis

Cells at a density of 1 × 10^6^ cells per well were plated on a six-well plate and then exposed to varying concentrations (50, 100 and 150 *μ*M) of furanodienone for 24 h. After treatment, both detached and attached cells were collected and centrifuged at 1000 r.p.m./min for 5 min at 4 °C. Washed twice with PBS, pelleted cells were fixed in 500 *μ*l 70% cold ethanol for at least 2 h. Before analysis using flow cytometry, cells were washed again with PBS and incubated with 100 *μ*l RNase. The resulting suspension was placed at 37 °C for 30 min. Subsequently, a volume of 400 *μ*l propidium iodide was added into the suspension, and cells were stained at 4 °C in the dark for 30 min. Cell cycles were detected at 488 nm using BD Accuri C6 and analysis were conducted with BD CFlow software (BD Biosciences, Mountain View, CA, USA).

### RNA isolation and real time RT-qPCR

The cell cycle regulatory gene expressions of P21^Cip1^, CDK 4, cyclin D1 and CDK 6 mRNA in RKO cells were measured by RT-qPCR analysis. Total RNA was extracted using Trizol reagent (Takara, Tokyo, Japan) according to the manufacturer’s instructions. RNA from each sample (1 *μ*g) was used to synthesize complementary DNA using RevertAid First Strand cDNA Synthesis Kit (Fermentas, Billerica, MA, USA) according to recommendations. RT-PCR was cycled between 95 °C for 15 s and 60 °C for 1 min for 40 cycles, after a 95 °C denaturation step for 5 min. Endogenous control was that of GAPDH to normalize the detected mRNA expressions. The primer pairs of P21^Cip1^, CDK 4, cyclin D1, CDK 6 and GAPDH were as follows: P21^Cip1^ (F) 5′-GACTGTGATGCGCTAATGGC-3′, (R) 5′-CCGTGGGAAGGTAGAGCTTG-3′ CDK 4 (F) 5′-CAGCTTGCCCGAGTTCTACT-3′, (R) 5′-TGTCCTCAGAGTTAGCCGGA-3′ cyclin D1 (F) 5′-CAGATCATCCGCAAACACGC-3′, (R) 5′-AAGTTGTTGGGGCTCCTCAG-3′ CDK 6 (F) 5′-AGGAAGGCAAACGTGACC-3′, (R) 5′-TATTGTCCCAAGGCTGGCTC-3′ GAPDH (F) 5′-GATGCCCCCATGTTCGTCAT-3′, (R) 5′-TCTTCTGGGTGGCAGTGATG-3′.

### DAPI staining

Cells were fixed with 4% paraformaldehyde for 30 min at room temperature. Washing with PBS, 100 *μ*l DAPI (Beyotime Biotechnology) was added to the fixed cells for 5 min and the cells were assessed by fluorescence microscopy (Leica, Wetzlar, Germany).

### Cell apoptosis by flow cytometry

The measurement of cell apoptosis was undertaken using an Annexin V-FITC/PI Apoptosis detection kit (BD Pharmingen, San Jose, CA, USA). Briefly, cells were plated on a six-well plate and then maintained at 37 °C in a humidified atmosphere with 5% CO_2_. After adherent culture, furanodienone in 0.1% DMSO or 5-FU was added into each well, for 24 h. Both floating and adherent cells were collected, washed twice with cold PBS and resuspended in 1 × binding buffer. The cells were incubated with 5 *μ*l Annexin V-FITC and 5 *μ*l PI for 15 min at room temperature in the dark. A 400 *μ*l 1 × binding buffer was added into each sample before being analyzed using flow cytometry (BD Accuri C6) with cell counts of 1 × 10^4^ for each measurement.

### Ethidium bromide and acridine orange staining for apoptosis detection

Confluent cell monolayers were exposed to furanodienone or 5-FU for 24 h at 37 °C. Washed with PBS for twice, cells were immediately treated with a dye mixture containing EB (100 *μ*g/ml) and AO (100 *μ*g/ml) in a 1:1 ratio for 5 min. Subsequently, cells were imaged using a florescence microscope equipped with a digital imaging system (Leica).

### Lactate dehydrogenase release assay

The cycotoxicity of furanodienone on human colon cells was assessed with LDH cytotoxicity Assay Kit (Jiancheng, Nanjing, China) according to the manufacture’s instructions.

### Assays for activation of caspase-3, -8, -9

Briefly, floating and attached cells were collected after washing twice with ice-cold PBS and they were resuspended in lysis buffer on ice for 30 min. Next, the lysate was centrifuged at 18 000 × *g* at 4 °C for 10 min and the supernatant was transferred to a 1.5 ml centrifuge tube. The total protein concentration of each sample in the supernatant was measured using the Bradford protein assay kit (Beyotime Institute of Biotechnology, Jiangsu, china). Subsequently, a mixture of 40 *μ*l detection buffer, 50 *μ*l cellular extracts and 10 *μ*l peptide substrates, respectively, Ac-DEVD-*p*NA, Ac-IETD-*p*NA and Ac-LEHD-*p*NA were co-incubated for 2 h at 37 °C in a 96-well plate. The release of *p*NA was measured at 405 nm with a spectrophotometer and the relative activity of caspase was calculated as the folds of treated cells to the control.

### Mitochondrial membrane potential assay

The loss of mitochondrial membrane potential, which is one of the early key events linked to apoptosis, was determined using the mitochondria-specific cationic fluorescence dye JC-1 (Beyotime Biotechnology). Treatment was carried out for 24 h, Cells were then suspended in 1 × JC-1 staining buffer for 30 min, co-incubating at 37 °C in the dark. The level of MMP was monitored by flow cyotometry for quantitative assessment. The red and green fluorescence were detected by FL-2 and FL-1 channels, respectively, using flow cytometer (BD Accuri C6), and the change in MMP was calculated as the folds of the green fluorescence intensity to that of the control.

### ATP determination

The intracellular ATP content exposure to furanodienone in RKO and HT-29 cells was determined quantitatively using the ATP assay kit (Beyotime Biotechnology) according to the manufacturer’s instructions.

### Measurement of intracellular ROS

The level of intracellular ROS was estimated quantitatively using an peroxide-sensitive fluorescent probe DCFH-DA (2′,7-dichlorofluorescein diacetate), which is oxidized in the presence of peroxides to the highly fluorescent DCF (2′,7-dichlorofluorescein).^51^ Cells plated in six-well plates (1 × 10^6^ cells per well) were exposed to furanodienone (150 *μ*M) for 24 h in the absence or presence of 10 mM NAC or 100 *μ*M z-VAD-fmk for 2 h at 37 °C in incubator. The cells were then co-incubated with 10 *μ*M DCFH-DA at 37 °C for 30 min before the fluorescent intensity of DCF was analyzed by flow cytometry (BD C6 Biosciences, San Jose, CA, USA) with an excitation wavelength of 488 nm and emission wavelength of 525 nm. The images of the stained cells were taken by inverted fluorescence microscopy immediately (Leica).

### Assessment of oxidative damage

After exposure to furanodienone with or without the pretreatment of 10 mM NAC for 24 h, cells were washed three times with cold PBS and then lysed in 500 *μ*l lysis buffer for 30 min on ice. Centrifuged at 150 000 × *g* for 3 min at 4 °C, the total concentration of proteins was determined using BCA protein assay kit (Beyotime Biotechnology). The lipid peroxidation product MDA and GSH level were measured by a micromount MDA Kit and glutathione Kit, both of which were provided from Nanjing Jiancheng Bioengineering Institute (Nanjing, China). The activities of SOD and CAT were analyzed using Total Superoxide Dismutase Assay Kit and Catalase Assay Kit (Nanjing Jiancheng Bioengineering Institute), respectively.

### Western blot analysis

The expression level of targeted protein was investigated by western blotting. Cell pellet was lysed in 100 *μ*l modified RIPA buffer for 30 min on the ice, and the concentration of total proteins was determined by a BCA protein assay kit (Beyotime Biotechnology). Proteins (30 *μ*g) for each sample were separated on 10–12% SDS-PAGE at 100 V for 1.5 h and transferred onto polyvinyldene difluoride membranes at 300 mA for 2 h afterward. The membranes were blocked with 5% (w/v) non-fat milk in Tris-buffered saline-Tween (TBST) buffer for 1.5 h at room temperature and then incubated with primary antibody at 4 °C overnight. Washed three times with TBST buffer, the blots were then incubated with HRP-conjugated secondary antibody for 1 h at room temperature. Each band was developed using enhanced chemiluminescence kit (Millipore, Billerica, MA, USA).

### Human CRC xenograft experiment

Twelve male BALB/c-nu mice (Shanghai Slac Laboratory Animal Co., Ltd, Shanghai, China) at 4 weeks of age were maintained in a standard animal laboratory supplied with sterilized water and food. RKO cells at a density of 5 × 10^6^/ml were suspended in cold PBS, and a volume of 150 *μ*l cell suspension was administrated subcutaneously in the right flank of each mouse. When the tumor size reached 50 mm^3^, the mice were randomly divided into two groups; control (10% DMSO) and furanodienone (2 mg) diluted with 10% DMSO. Both groups received intraperitoneal injection every other day for seven times. The tumor size was monitored every 2 days for a period of 4 weeks with a sliding caliper and calculated using the formula: length × width^2^/2. At the end point of the experiment, the mice were killed and the tumor tissues were removed from the mice, and fixed in 5% formalin for the next immunohistochemistry, HE and TUNEL assays. All the animal-related procedures were approved by the Animal Care and Use Committee of Shanghai General Hospital, Shanghai, China.

### TUNEL assay

Apoptosis detection in the tumor tissues was identified using the terminal deoxynucleotidyl transferase-mediated dUTP nick end labeling (TUNEL) assay with *In Situ* Cell Death Detection Kit, Fluorescein (Roche Diagnostics, Mannheim, Germany). Formalin-fixed tumor tissues were embedded in paraffin and cut into serial sections (4 *μ*m). In brief, after being deparaffinized with xylene and ethanol, and hydrated proteinase K (20* μ*g/ml) for 25 min at 37 °C, slides were washed with PBS for several times and then incubated with TUNEL reaction mixture prepared freshly for 1 h at 37 °C in a moist chamber. After being again washed, the apoptotic cells on the slides were observed under fluorescence microscopy (Leica).

### Histopathology and immunohistochemistry

Following a hydration process, the slides were exposed to hematoxylin for 15 min and immersed in 1% hydrochloric acid in 75% ethanol for 30 s. The slides were stained with eosin for 5 min followed by dehydration. Finally, the slides were immersed in xylene and mounted. For immunohistochemical staining, the slides were deparaffinized in xylene and rehydrated with graded alcohol and incubated in 3% H_2_O_2_ to block the endogenous peroxidase activity. Antigen retrieval was performed in 10 mM sodium citrate buffer (pH 6.0) for 30 min by boiling the slides. Then, slides were blocked in 10% normal goat serum for 15 min, followed by incubation with cleaved caspase-3, p-JNK, p-P38 and p-ERK at 4 °C overnight in a moist chamber. Afterward, slides were washed in PBS for three times and then incubated with the second antibody for 30 min at room temperature. Immunoreactivity was visualized using the Vectastain Elite DAB KIT (Vector Laboratories, Burlingame, CA, USA). Image Pro-Plus 6.0 (IPP, Media Cybernetics, BD Biosciences) was used for digital analysis.

### Statistical analysis

All data were repeated at least three times independently for statistical calculations and were expressed as mean±S.D. Statistical significance (*P<0.05, **P<0.01 and ***P<0.0001, compared to the control group) was determined by GraphPad Prism 5 (GraphPad Software, Inc., La Jolla, CA, USA) using one-way ANOVA analysis or Student’s *t*-test.
